# Physiological Characteristics of *Lactobacillus casei* Strains and Their Alleviation Effects against Inflammatory Bowel Disease

**DOI:** 10.4014/jmb.2003.03041

**Published:** 2020-05-31

**Authors:** Yang Liu, Yifeng Li, Xinjie Yu, Leilei Yu, Fengwei Tian, Jianxin Zhao, Hao Zhang, Qixiao Zhai, Wei Chen

**Affiliations:** 1 State Key Laboratory of Food Science and Technology, Jiangnan University, Wuxi, Jiangsu 2422, P. R. China; 2 School of Food Science and Technology, Jiangnan University, Wuxi, Jiangsu 141, P.R. China; 3 Hwa Chong Institution (College), 661 Bukit Timah Road, Singapore 26974, Singapore; 4 International Joint Research Laboratory for Probiotics at Jiangnan University, Wuxi, Jiangsu 21122, P.R. China; 5 National Engineering Research Center for Functional Food, Jiangnan University, Wuxi, Jiangsu 214122, P.R. China; 6 Wuxi Translational Medicine Research Center and Jiangsu Translational Medicine Research Institute, Wuxi Branch, P.R. China; 7 (Yangzhou) Institute of Food Biotechnology, Jiangnan University, Yangzhou 225004, P.R. China; 8 Beijing Innovation Center of Food Nutrition and Human Health, Beijing Technology and Business University (BTBU), Beijing 10004, P.R. China

**Keywords:** *Lactobacillus casei*, physiological characteristic, probiotic, inflammatory bowel disease, gut microbiota, NF-κB

## Abstract

*Lactobacillus casei*, one of the most widely used probiotics, has been reported to alleviate multiple diseases. However, the effects of this species on intestinal diseases are strain-specific. Here, we aimed to screen *L. casei* strains with inflammatory bowel disease (IBD)-alleviating effects based on in vitro physiological characteristics. Therefore, the physiological characteristics of 29 *L. casei* strains were determined, including gastrointestinal transit tolerance, oligosaccharide fermentation, HT-29 cell adhesion, generation time, exopolysaccharide production, acetic acid production, and conjugated linoleic acid synthesis. The effects of five candidate strains on mice with induced colitis were also evaluated. The results showed that among all tested *L. casei* strains, only *Lactobacillus casei* M2S01 effectively relieved colitis. This strain recovered body weight, restored disease activity index score, and promoted anti-inflammatory cytokine expression. Gut microbiota sequencing showed that *L. casei* M2S01 restored a healthy gut microbiome composition. The western blotting showed that the alleviating effects of *L. casei* M2S01 on IBD were related to the inhibition of the NF-κB pathway. A good gastrointestinal tolerance ability may be one of the prerequisites for the IBDalleviating effects of *L. casei*. Our results verified the efficacy of *L. casei* in alleviating IBD and lay the foundation for the rapid screening of *L. casei* strain with IBD-alleviating effects.

## Introduction

Inflammatory bowel disease (IBD) is an intestinal disease that mainly presents as Crohn’s disease (CD) and ulcerative colitis (UC). Common clinical symptoms include diarrhea, abdominal pain, and bloody stools in severe cases. Although IBD has only emerged in the past 150 years, the incidence of IBD in many countries and regions is rising. Currently, IBD prevalence in partially developed countries exceeds 0.3% [1]. In the past 30 years, the incidence of IBD in South America has been rising. In therazil had the fastest rate of increase, with a UC prevalence of 75% and a CD prevalence of 117% [2]. In Asia, the average annual incidence of IBD is 1.50 per 100,000 people. India and China have the highest incidences of IBD, at 9.31 and 3.64 per 100,000 people, respectively [3].

Probiotics are receiving attention as a potential treatment for IBD, and strains including *Bacillus licheniformis* [4], *Bacteroides fragilis* [5], *Bifidobacterium longum* [6], *Lactococcus lactis* [7], *Lactobacillus plantarum* [8], *Lactobacillus reuteri* [9] and many others have been shown to restore body weight, regulate the gut microbiome and restore intestinal barrier integrity in mouse models. This indicates the potential of probiotics to significantly prevent or inhibit the occurrence and development of IBD. In clinical trials, several strains of *Lactobacillus* and *Bifidobacterium* have shown therapeutic effects on IBD, including alleviation of intestinal mucosal inflammation, reduction of colonic myeloperoxidase, and fecal calprotectin levels, and increased time between symptom recurrences [10-13]. *Lactobacillus casei* (*L. casei*) is known to exert health-promoting functions and is widely found in functional foods. Several strains of *L. casei*, such as Shirota, BL23 and ATCC 393, have been reported to mitigate damage caused by intestinal diseases by improving disease activity index (DAI) score, restoring histopathological damage [14], inhibiting pro-inflammatory cytokine expression [15] and NF-κB signaling [16], and promoting the differentiation of Treg cells [17]. These findings indicate that *L. casei* strains have the potential to alleviate intestinal diseases. However, other studies have shown *L. casei* is limited in the treatment of intestinal diseases. Ingestion of *L. casei* did not inhibit the occurrence of diarrhea or prevent pathogenic infections [18, 19]. These conclusions suggest that the effects of *L. casei* on intestinal diseases are strain-specific.

Previous research has found that the beneficial effects of probiotics relate to their physiological characteristics. A strain’s adhesion ability and its ability to tolerate gastrointestinal transit are directly related to its colonization of the gastrointestinal tract [20]. Effective fermentation of oligosaccharides can promote cell proliferation and resistance to intestinal injury [21]. Probiotic metabolites, such as exopolysaccharides (EPSs) and short-chain fatty acids (SCFAs), are also implicated in immune regulation [22], intestinal motility [23], intestinal mucosal barrier protection [24] and antagonization of pathogenic bacteria [25]. IBD-related studies also indicate that the physiological characteristics of probiotic strains may contribute to the alleviation of IBD: microencapsulation was found to significantly improve the gastrointestinal transit tolerance of *Lactobacillus rhamnosus* GG, and thus improved its colitis-alleviating effects [26]. Supplementation of *Bacillus coagulans* MTCC5856 with prebiotics was more effective than *Bacillus coagulans* MTCC5856 alone in the treatment of IBD [27]. The EPS and conjugated linoleic acid (CLA) produced by probiotics were also shown to restore intestinal mucosal barrier function and prevent colitis [28]. These studies indicate that the physiological characteristics of probiotics are related to their IBD-alleviating effects. However, previous studies only measured a single physiological characteristic to screen candidate strains from multiple species. Few studies have measured multiple physiological characteristics in a single species. It is still unknown what kind of physiological characteristics would contribute to the IBD-alleviating effects of *L. casei* strains.

Therefore, in this study, seven physiological characteristics of *L. casei* strains, including gastrointestinal transit tolerance, oligosaccharide utilization, HT-29 cell adhesion, generation time, EPS production, acetic acid production, and CLA synthesis were examined to screen several candidate strains with different typical physiological characteristics. Then the effects of candidate strains on IBD indicators, including body weight, DAI score, colon length restoration, gut microbiome regulation, anti-inflammatory factor expression, inhibition of NF-κB signaling and histopathological signs of recovery, were evaluated in mice, with the aim of finding *L. casei* strains with IBD-alleviating effects.

## Materials and Methods

### Bacterial Strains and Culture

All *L. casei* strains, including RS8-5, RS29-1, JS-WX-3-L-3, RS42-2, 5-1L, MJ1, NT52-4, NT72-1, HN13-1, 104S2, M2-03-F02-L4-1-5 (M25), M2-06-F01-L4-2-4, GD41-6, 34-3, 35-7, FJSSZ4-L2, F-FJND-D7-M5, V-GZTR-132-M8, V-CQYB7-171-M7, V-CQYB6-170-M3 (VM3), F-JS-CZ-D2-L-3, FJSWX33-L3, V-CQRC7-161-M2, M2-01-R02-S01 (M2S01), F-ZJHZ-D2-M1, V-CQQJ4-174-M3, V-CQQJ3-173-M1, V-CQYoY1-157-M2 (VM2), and CCFM30, were provided by the Culture Collections of Food Microbiology, Jiangnan University (China) and cultured in MRS broth for 20 h at 37°C.

### Gastrointestinal Transit Tolerance Assay

The gastrointestinal transit tolerance of *L. casei* was measured according to the method of Maragkoudakis *et al*.[29]. Pepsin (1:10,000, Sangon Biotech Ltd., China) was suspended in 0.5% w/v sterile saline at 3 g/l and the pH adjusted to 3.0 to simulate gastric juices. In this simulated gastric juice, the bacterial incubation time was 180 min. Trypsin (1:250, Sinopharm Chemical Reagent Ltd., China) and bile salt (LP0055, Thermo Fisher Scientific, UK) were dissolved in 0.5% w/v sterile saline at a concentration of 1 g/l and 3 g/l, respectively, then adjusted to pH 8.0 to simulate small intestinal juices. In the simulated small intestinal juices, the bacterial incubation time was 240 min. The viable counts of *L. casei* were determined by plate counting at 0 and 420 min (180 min plus 240 min). The gastrointestinal transit tolerance of *L. casei* strains was determined by comparing the number of viable cells at different time points.

### FOS and GOS Fermentation Assay

The fructooligosaccharide (FOS) (95% purity, Baolingbao Biology Ltd., Dezhou, China) and galactooligo-saccharide (GOS) (95% purity, Quantum Hi-Tech Ltd., China) fermentation ability of *L. casei* was measured according to the method of Kaplan and Hutkins [30], with modiﬁcations. Modified MRS agar was used as the experiment media. Briefly, 0.0075% (w/v) bromocresol purple (Sinopharm Chemical Reagent Ltd.) and 0.3% FOS (or GOS) were added to the MRS medium, which did not contain glucose or meat extract. The pH was adjusted to 7.2. MRS medium without glucose, meat extract, or FOS/GOS was used as the control. Each cultivated strain was diluted and spread on experimental media plates and control media plates and incubated for 12 h at 37°C. The FOS and GOS fermentation abilities of *L. casei* strains were determined by whether or not a yellow zone surrounding the colonies appeared.

### Adhesion Ability Assay

The adhesion ability of *L. casei* was measured according to the method of Walsham *et al*. [31], with modiﬁcations. Briefly, HT-29 cells were cultured in RPMI 1640 media (Thermo Fisher Scientific Co.) supplemented with 10%heat-inactivated fetal bovine serum (Thermo Fisher Scientific Co.) and 20 mg/ml of streptomycin and penicillin (Bio-Light Biotechnology Ltd., China). The culture conditions for the cells were 37°C and a 5% CO_2_ atmosphere. Cells were seeded on 6-well plates at a density of 1 × 10^5^ cells/ml and cultivated for 12 h. Bacteria were harvested by centrifugation (8,000 ×*g*, 4°C, 5 min) and washed twice with sterile PBS. The cells were inoculated with a ﬁnal concentration of 5×10^7^ CFU/ml in RPMI 1640 medium. After 120 min incubation, the cells were washed three times with sterile PBS. Six-well tissue culture plates were examined under a microscope (BA410E microscope, Motic China Group Ltd., China), and the number of bacteria adhering to 100 cells were counted. The adhesion ability of *L. casei* was expressed as bacteria per cell.

### Generation Time Assay

The generation time of *L. casei* was measured according to the method of Shi *et al*. [32]. To determine generation time, all *L. casei* strains were cultured for 600 min. The 600 nm optical density of the bacterial culture medium was recorded every 120 min using a Multiskan GO microplate reader (Thermo Fisher Scientific Co.). The generation time (G) was calculated by:

(1) $$P_{t}=P_{0}\times 2^{n}$$

(2) $$n=\log P_{t}-\log P_{0}/\log2$$

(3) G=t/n

P_0_ and *P*_t_ indicate the initial concentration of bacteria and the concentration at the end of the selected period, *t* represents time and n represents the number of generations.

### EPS Production Assay

The EPS production ability of *L. casei* was measured according to the method of Tallon *et al*. [33]. EPSs produced by *L. casei* strains were quantified using the phenol/sulfuric acid method. Glucose was used to generate a standard curve. The results were expressed in mg (glucose)/g (dry cell weight).

### Acetic Acid Production Assay

Bacterial culture medium (0.5 ml) was acidified using sulfuric acid (10%), and 0.8 ml ether was added to the solution to extract acetic acid. The acetic acid production of *L. casei* was measured by GC-MS, and the conditions for GC-MS referred to Wang *et al*. [34].

### CLA Synthesis Ability Assay

The CLA synthesis ability of *L. casei* was measured per the method of Yang *et al*. [35]. All strains were cultured in MRS broth containing linoleic acid (0.5 mg/ml) in an anaerobic environment for 72 h. After 72 h, the supernatant was separated by centrifugation (5,000 ×*g*, 4oC, 10 min), and CLA was extracted by adding normal hexane, followed by a methylation process. The conversion rate of CLA were measured by GC-MS.

### Animal Experiment Design

Adult male SPF C57BL/6 mice weighing 26-28 g (10 weeks, Shanghai Laboratory Animal Center, China) were housed in a 12 h light/dark cycle environment under controlled temperature (22-24°C) and humidity (40-70%). The mice were fed standard commercial mouse food and distilled water. All animal experiments were approved by the Ethics Committee of Jiangnan University, China (JN.No20171115c2401220[73]) and were carried out under the guidelines set by the European Community (Directive 2010/63/EU).

Seventy mice were randomly assigned to stainless steel cages with 5 animals per cage (two cages per group) and were acclimatized for one week before the experiment. With the exception of the control group, groups were given 3.5% (w/v) dextran sulfate sodium (DSS, 36-50 kDa, MP Biomedicals Ltd., USA) in drinking water for 7 consecutive days to induce colitis. During this period, control group and DSS group mice were administered sterile PBS (0.2 ml/mouse/day) whereas other groups were administered *L. casei* (1×10^9^ CFU/0.2 ml/mouse/day) strain suspension by gavage, as follows:

(1) Control group: Distilled water + sterile PBS for 7 days.
(2) DSS group: DSS + sterile PBS for 7 days.
(3) *L. casei* group 1: DSS + *L. casei* M25 bacterial suspension for 7 days.
(4) *L. casei* group 2: DSS + *L. casei* VM3 bacterial suspension for 7 days.
(5) *L. casei* group 3: DSS + *L. casei* M2S01 bacterial suspension for 7 days.
(6) *L. casei* group 4: DSS + *L. casei* VM2 bacterial suspension for 7 days.
(7) *L. casei* group 5: DSS + *L. casei* CCFM30 bacterial suspension for 7 days.

During the experiment, the body weight, stool characteristics, presence of blood in stool and DAI score were monitored and calculated daily. The DAI score was calculated by the formula:

DAI = (body weight loss index + stool consistency index + stool blood content index)/3.

Body weight loss index: body weight loss <1%, 0 points; <5%, 1 point; <10%, 2 points; > 15%, 4 points.

Stool consistency index: normal, 0 points; loose, 2 points; diarrhea, 4 points.

Stool blood content index: no occult blood, 0 points; occult blood, 2 points; clearly visible blood, 4 points.

After 7 days of gavage, fresh stool samples were collected and stored at -80°C. The mice were sacriﬁced. Their colons were separated and measured. Portions of colon were fixed with paraformaldehyde while the remainder was stored at -80°C.

### Histological Evaluation

Colon samples were dehydrated in ethanol, followed by sectioning (5 μm) and paraffin embedding, before staining with hematoxylin and eosin. The histological damage to the colon was examined with a microscope (BA410E microscope, Motic China Group Ltd.) and evaluated based on the colonic epithelium injury, villi injury, inflammatory cell infiltration degree, and edema degree. The scoring criteria are: no obvious symptoms, 0 points; moderate symptoms, 0.5 points; severe symptoms, 1 points. The histopathology score is the sum of the sub-scores.

### Biochemical Analysis of Colon

Colon samples (0.10 g) were homogenized in ice-cold PBS (pH 7.4), then centrifuged (3,000 ×*g*, 4°C, 5 min). The IL-10 and IL-22 levels in the colon were measured with assay kits (SenBeiJia Biological Technology Ltd., China).

### Fecal DNA Extraction and Illumina Miseq Sequencing

Fecal bacterial DNA was extracted using a FastDNA Spin Kit (MP Biomedicals Ltd.) according to the manufacturer’s instructions. PCR and sequencing of gut microbiota composition were performed according to the method described by Wang *et al*. [36]. The forward primer sequence and the reverse primer sequence of the V3-V4 region were 5′-CCTAYGGGRBGCASCAG-3′ and 5′-GGACTACNNGGGTA-3′. The QIIME 2 was used to analyze the data. Operational taxonomic units (OTUs) were chosen to 97% identity against the silva database (v 13_8). With a minimum number of 7706 reads, the sequences of each sample were randomly read to normalize the data, which were used in the diversity analyses. Principal component analysis (PCA) analysis was conducted with STAMP software, and LEfSe analysis was used to analyze differences in gut microbiota composition.

### Western Blotting Analysis

Colon samples were lysed in radio immunoprecipitation assay lysis buffer (P0013K, without inhibitors, Beyotime Biotechnology Ltd., China) containing protease and phosphatase inhibitor mixtures (50×, Beyotime Biotechnology Ltd.), then centrifuged (14,000 ×*g*, 4°C, 15 min) to collect the supernatant. The total concentration of protein was measured using a BCA protein assay kit (Beyotime Biotechnology Ltd.). Equal amounts of protein were separated using 8% SDS-PAGE and subsequently transferred to PVDF membranes, followed by a blocking process (60 min, room temperature). Five percent BSA (w/v) in Tris-buffered saline Tween 20 (0.1%, v/v) was used for blocking. The membrane was incubated with antibodies against NF-κB-p65 (ab16502, 1:2000, Abcam Ltd., USA), p-p65 (ab86299, 1:2000, Abcam Ltd.) and β-actin (1:1000, Santa Cruz Biotechnology Co., USA) at 4°C for 16 h. The membrane was washed and incubated with corresponding secondary antibodies (A00098 and A00160, GenScript Biotechnology Ltd., China) for 60 min at room temperature. Following incubation, the membrane was washed and visualized using ECL western blotting detection reagent (ProteinSimple Co., USA). Alpha View Fluorchem Fc3 software (ProteinSimple Co.) was used to analyze the bands.

### Statistical Analysis

All data are expressed as mean ± standard error of the mean (SEM). One-way analysis of variance (ANOVA) was used to analyze the results, followed by Tukey’s multiple comparison test to determine statistical signiﬁcance. *p*-values less than or equal to 0.05 were considered to be statistically significant.

## Results

### Gastrointestinal Transit Tolerance of *L. casei* Strains

**Fig. 1:**
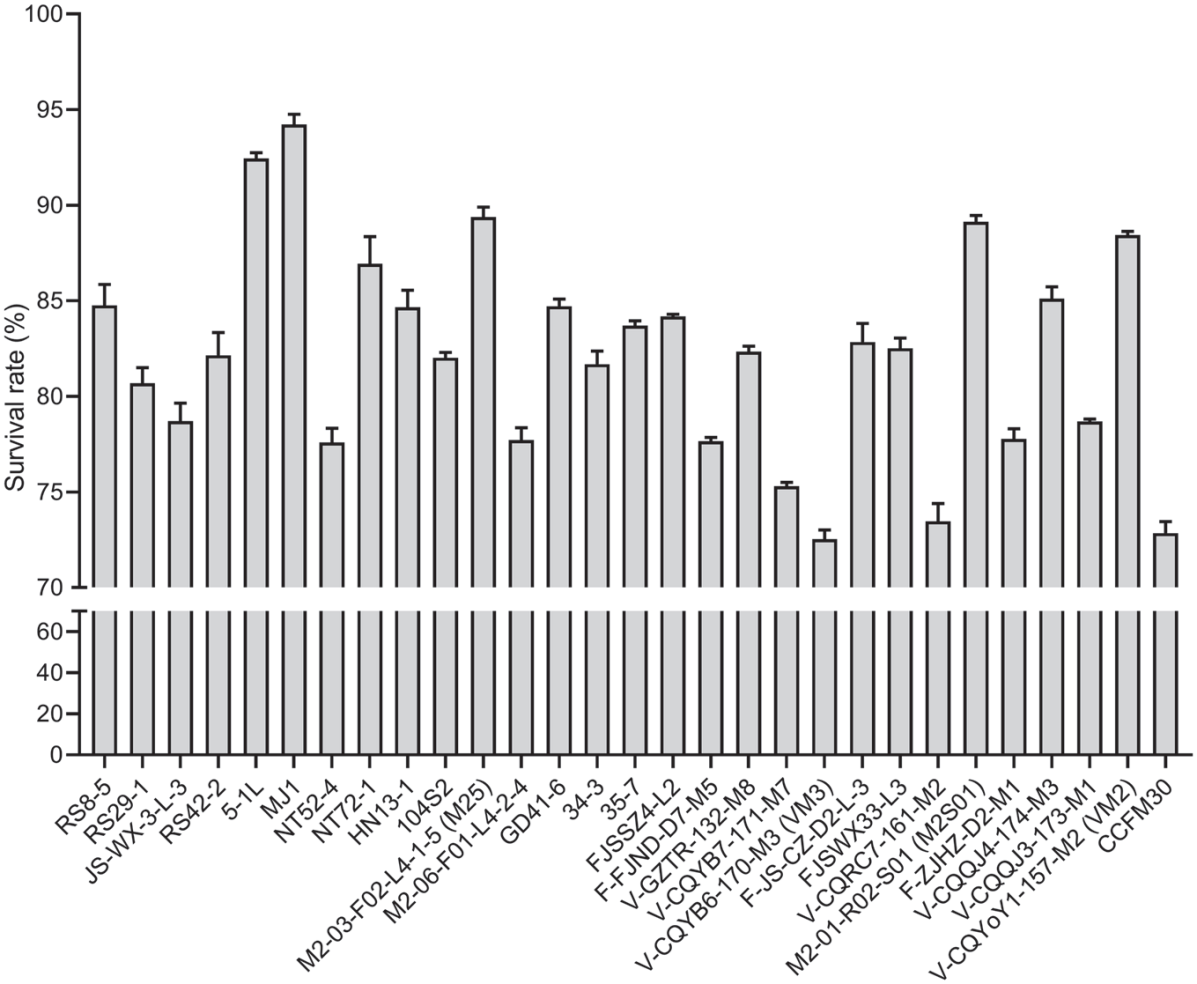
The survival rate of *Lactobacillus casei* strains following in vitro simulated gastrointestinal transit. The viable counts of *L. casei* strains were determined by plate counting at 0 and 7 h. The specific values of viable bacteria at the two time-points represent the gastrointestinal transit tolerance.

The survival rate of 29 *L. casei* strains following exposure to simulated gastric juices and simulated small intestinal juices fluctuated between 72% and 95% (Fig. 1). Strains with a survival rate higher than 85% or less than 75% were selected for GOS and FOS fermentation testing.

### GOS and FOS Fermentation by *L. casei* Strains

Table 1 shows the FOS and GOS utilization of 10 *L. casei* strains. The results indicate that FOS can be utilized by more strains than GOS. Two strains, VM2 and CCFM30, could ferment both kinds of oligosaccharides. M25, M3, and M2S01 could not ferment either oligosaccharide. Strains that could ferment both FOS and GOS or could not ferment either oligosaccharide were selected to test HT-29 cell adhesion, generation time, EPS production, acetic acid production, and CLA synthesis.

### Adhesion Ability of *L. casei* Strains

**Fig. 2:**
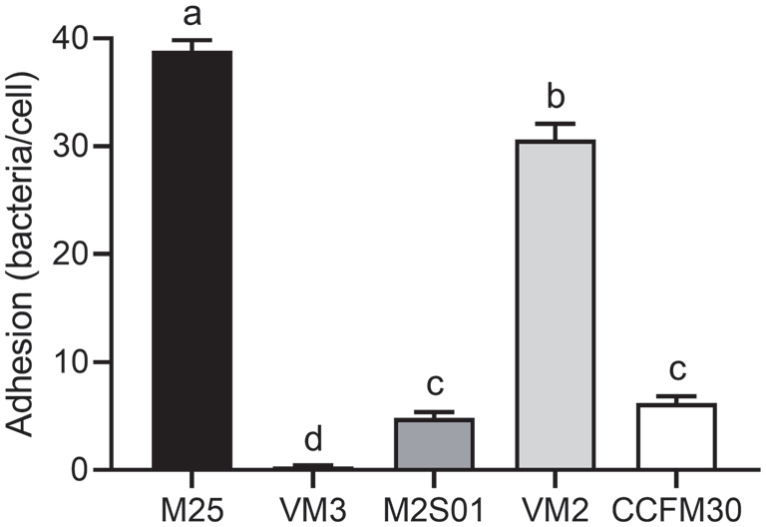
The average number of bacteria adhered to 100 cells counted by a microscope. Letters a to d indicate statistically significant differences (*p* < 0.05).

The adhesion ability of the different *L. casei* strains exhibited a polarization trend (Fig. 2, *p* < 0.05); the adhesion of two strains, M25 and VM2, exceeded 30 bacteria per cell, while VM3 exhibited the lowest adhesion with only 0.35 bacteria present per cell.

### The Generation Time, EPS Production, Acetic Acid Production, and CLA Synthesis of *L. casei* Strains

**Fig. 3:**
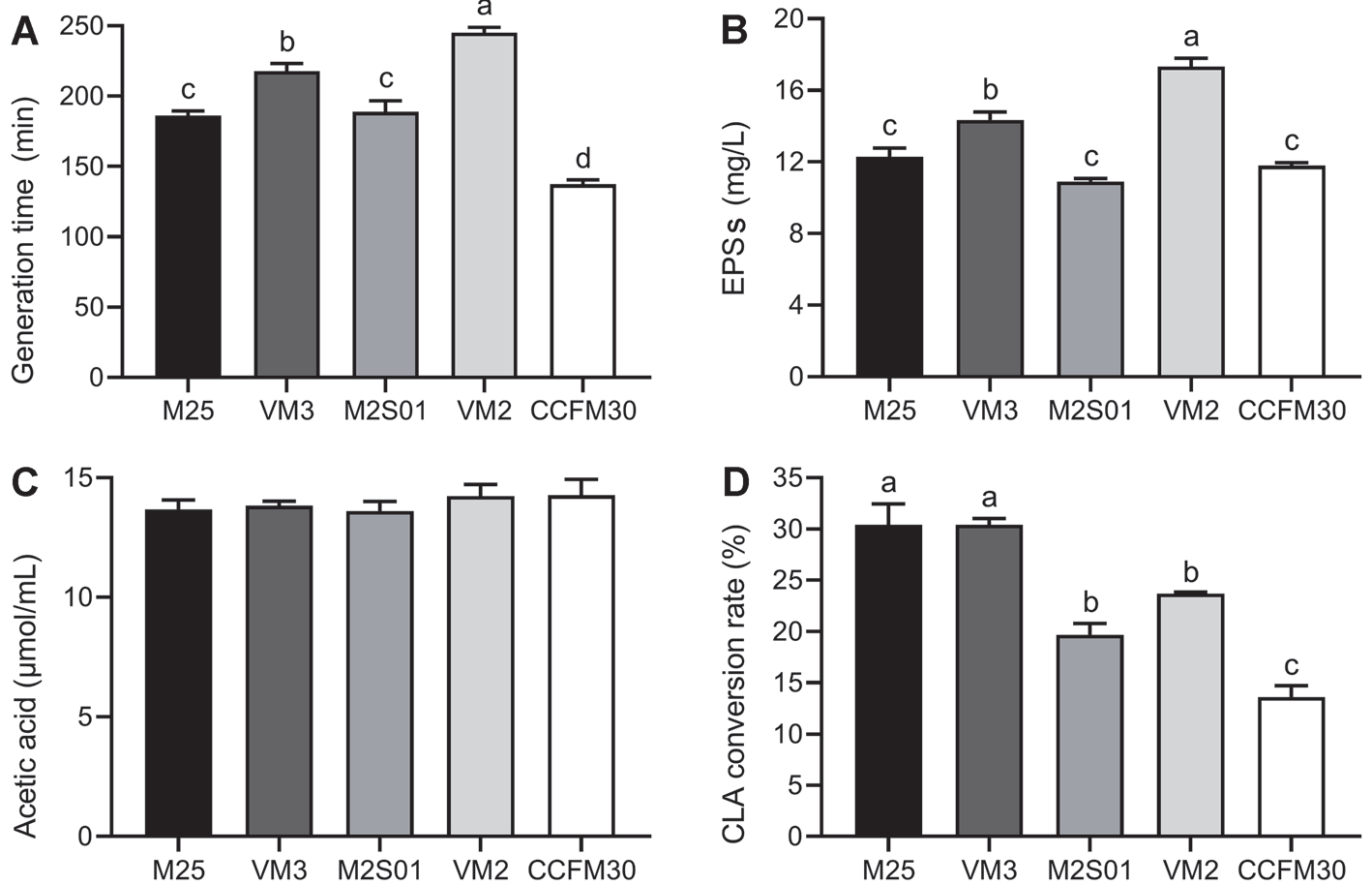
Generation time, EPS production, acetic acid production, and CLA conversion rate of *Lactobacillus casei* strains. (**A**) The ratio of time to the number of generations in the logarithmic period of the strain; (**B**) EPSs of *L. casei* strains quantified by phenol/sulfuric acid method; (**C**) Acetic acid produced by *L. casei* measured by gas chromatography-mass spectrometry; (**D**) The CLA conversion rate of *L. casei* measured by GC-MS. EPSs: exopolysaccharides; CLA: conjugated linoleic acid. Letters a to d indicate statistically significant differences (*p* < 0.05).

The generation time of the strains varied greatly, ranging from 130 min to 250 min (Fig. 3A). The fastest growing strain was CCFM30, with a generation time of 137.4 mins. VM2 was the slowest growing strain, with a generation time of 245.2 min. As shown in Fig. 3B, VM2 had the highest EPS production (*p* < 0.05), reaching 17.33 g/l. The lowest EPS producer was M2S01, producing 10.92 g/l. The EPS production of M25 and CCFM30 was slightly higher than M2S01. The EPS production of VM3 was lower than that of VM2 but higher than the other three strains (*p* < 0.05). The acetic acid production of all strains was approximately 400 μmol/ml. Quantifying the CLA conversion rate of each *L. casei* strain using GC-MS revealed that the abilities of different *L. casei* strains to synthesize CLA were significantly different (Fig. 3D, *p* < 0.05). Conversion rates ranged from 30% to 13%.

### Body Weight, DAI Score and Colon Length in Colitis-Induced Mice

**Fig. 4:**
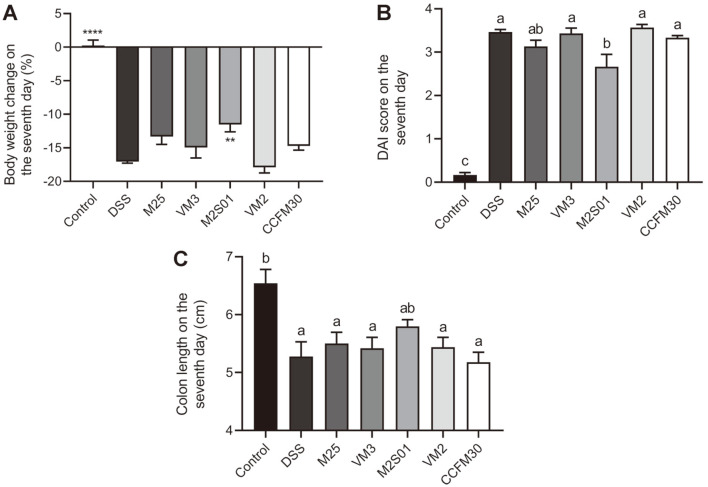
The effect of *Lactobacillus casei* strains on a DSS-induced colitis mouse model. (**A**) The percentage of weight loss after seven days of DSS intake (%); (**B**) DAI score after seven days of DSS intake. DAI score was calculated by the formula DAI = (body weight loss index + stool consistency index + stool blood content index)/3; (**C**) Colon length after seven days of DSS intake. **p < 0.01 vs DSS group, ****p < 0.0001 vs DSS group. Letters a, b and c indicate statistically significant differences (*p* < 0.05). DSS: dextran sulfate sodium; DAI: disease activity index.

The ingestion of DSS resulted in a significant drop in body weight, a rapid shortening in colon length and an increase in DAI (Figs. 4A-4C). Seven days post-induction, compared to the control group, these three variables showed significant changes in the DSS group (*p* < 0.05). Following oral administration of five different *L. casei* strains, only M2S01 attenuated body weight loss, increased colon length and recovered the DAI score (*p* < 0.05). These ﬁndings indicate that the M2S01 strain can more effectively prevent colitis-induced damage compared to other strains.

### Composition of Gut Microbiota

**Fig. 5:**
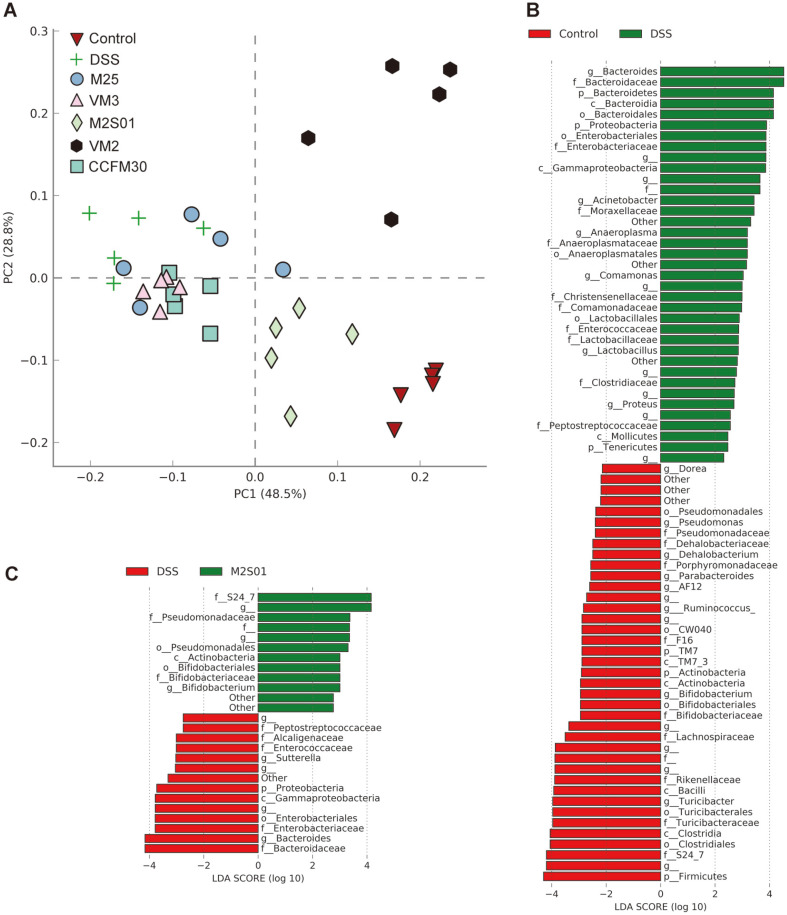
Gut microbiota composition in colitis-induced mice. (**A**) Principal component analysis of mouse gut microbiota; (**B**) LEfSe analysis of control group and DSS group; (**C**) LEfSe analysis of M2S01 group and DSS group. DSS: dextran sulfate sodium.

PCA analysis of gut microbiota composition in mice is shown in Fig. 5A. Following ingestion of DSS, the relative abundance of *Bacteroides, Acinetobacter, Anaeroplasma, Comamonas*, *Lactobacillus*, and *Proteus* increased significantly. *Dorea, Pseudomonas, Dehalobacterium, Parabacteroides, Ruminococcus*, *Bifidobacterium*, and *Turicibacter* decreased in abundance to varying degrees (Fig. 5B). Compared to the other four strains of *L. casei*, M2S01 had a superior effect on microbiota recovery, with an increase in *Bifidobacterium* and a decrease in Bacteroides (Fig. 5C). The species composition of the M2S01 group was closer to that of the control group than to that of the DSS group. Oral administration of *L. casei* M2S01 resulted in a more healthy gut microbiome.

### Anti-Inflammatory Cytokines and NF-κB Expression in the Colon

**Fig. 6:**
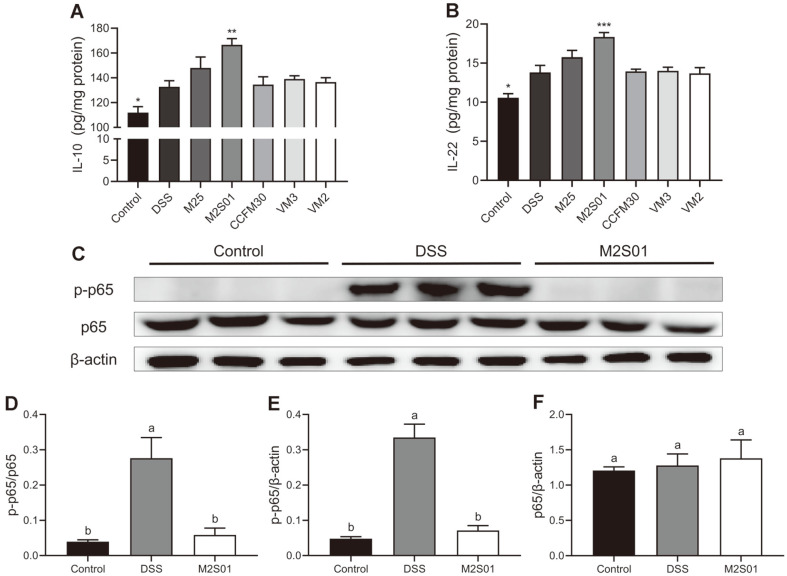
The effects of *Lactobacillus casei* on the expression of anti-inflammatory cytokines and NF-κB pathway in the colon. (**A**) The expression of IL-10 determined by the corresponding ELISA kits after seven days of DSS intake; (**B**) The expression of IL-22 determined by the corresponding ELISA kits after seven days of DSS intake; (**C**) The expression of p-p65 and p65 quantified by western blotting after seven days of DSS intake; (**D**) The relative gray value of p-p65 and p-65; (E) The relative gray value of p-p65 and β-actin; (F) The relative gray value of p65 and β-actin. *p < 0.05 vs DSS group, **p < 0.01 vs DSS group, ****p < 0.001 vs DSS group. Letters a and b indicate statistically significant differences (*p* < 0.05). DSS: dextran sulfate sodium.

The anti-inflammatory effects of five *L. casei* strains were evaluated by measuring the levels of anti-inflammatory cytokines IL-10 and IL-22 (Figs. 6A and 6B). The levels of IL-10 and IL-22 in the colon of the DSS group were significantly increased due to the presence of DSS in the drinking water. Compared to the DSS group, the *L. casei* M2S01 group had higher levels of IL-10 and IL-22 (*p* < 0.05). No therapeutic effects were observed for the other four *L. casei* strains. As seen in Figs. 6C ando differences in the expression of p65 were observed between the experimental groups. However, the p-p65 expression in the DSS group was significantly higher than the control group (Figs. 6C and 6E). The supplementation of *L. casei* M2S01 reduced the phosphorylation levels of p65 dramatically, to levels similar to that of the control (Fig. 6D). These results indicate that treatment with the *L. casei* strain M2S01 could prevent intestinal inflammation, and *L. casei* M2S01 might be able to regulate the NF-κB pathway, thereby inhibiting inflammation.

### Histopathological Analysis of Colon Tissue

**Fig. 7:**
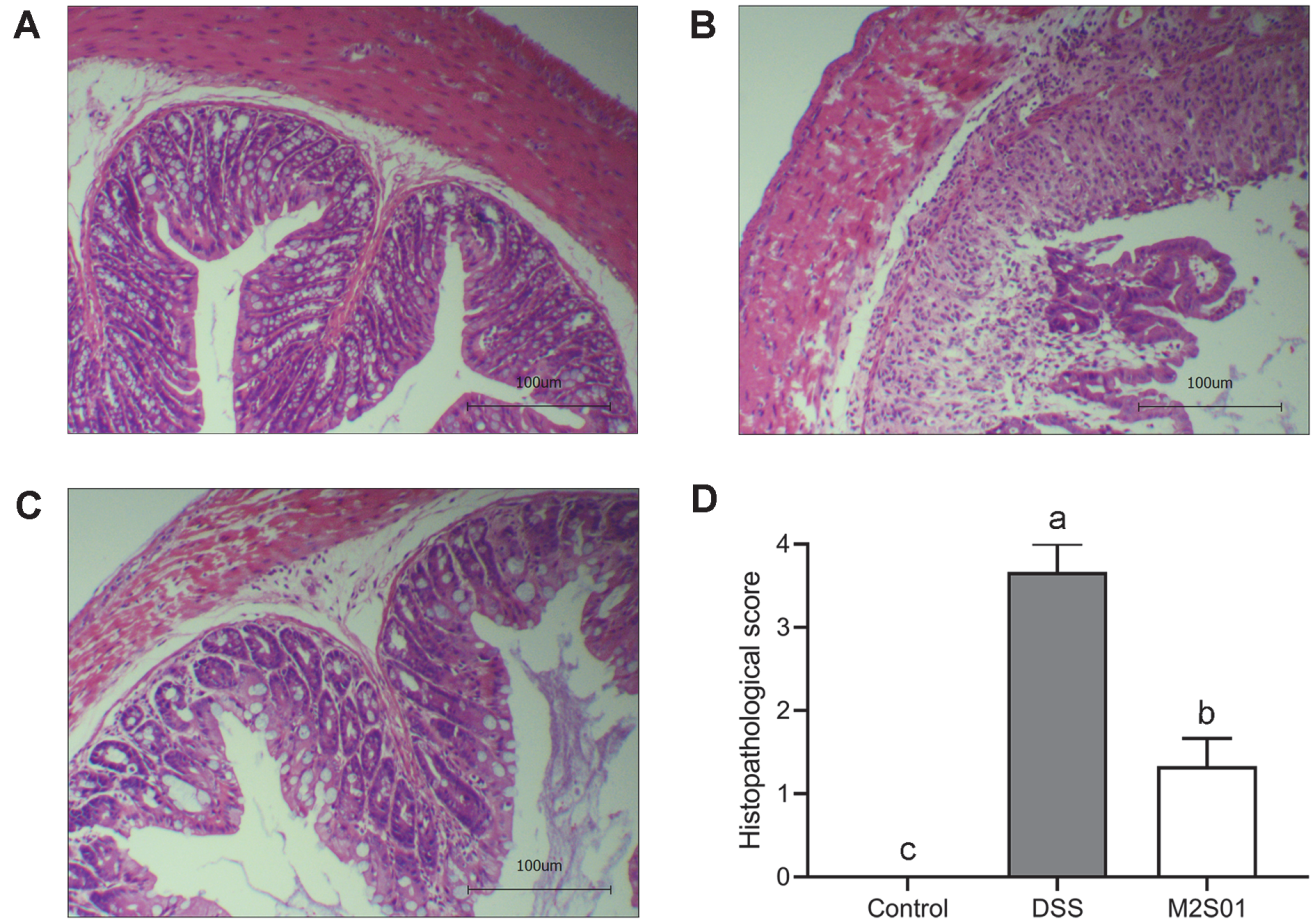
Histopathological analysis of colon tissue. (**A**) Histopathological analysis of the control group without DSS intake; (**B**) Histopathological analysis of the DSS group after seven days of DSS intake; (**C**) Histopathological analysis of the M2S01 group after seven days of DSS intake (100×; scale bar = 100 μm); (**D**) Histopathological score of each group. DSS: dextran sulfate sodium.

Histopathological analysis fully demonstrated the potential anti-inflammatory effects of *L. casei* M2S01. In the DSS group, almost all intestinal villi were missing. Intestinal lamina propria and intestinal submucosal fibroblasts were severely proliferated, accompanied by increased inflammatory cell infiltration (Figs. 7A and 7B). Treatment with *L. casei* M2S01 significantly reduced these signs of damage. Intestinal integrity was also restored (Fig. 7C). Moreover, the histopathological score showed that the colon injury in M2S01 group was significantly lower than that in DSS group (Fig. 7D).

## Discussion

In this study, we aimed to find L.casei strains with IBD-alleviating effects based on physiological characteristics. Therefore, seven IBD-related physiological characteristics of *L. casei* strains, including gastrointestinal transit tolerance, oligosaccharide fermentation, HT-29 cell adhesion, generation time, EPS production, acetic acid production, and CLA synthesis, were investigated in vitro. Five L.casei strains with different physiological characteristics were screened. The effects of these strains on IBD in animal models were also evaluated. A bacterial strain’s ability to survive gastrointestinal transit is necessary for colonization of the gut as well as for health-promoting effects. During the process of colonization, the metabolic processes, cell structure and gene expression of *Lactobacillus* change [37]. A strong tolerance of gastrointestinal transit can aid the strain in adapting to the intestinal environment, achieving colonization of the gut and exerting a protective barrier function, thereby preventing the occurrence of IBD [18]. The metabolic activity of probiotics in the colon is directly related to their health-promoting effect. FOS and GOS are two widely used oligosaccharide prebiotics that are mostly digested in the colon and have the ability to regulate gut microbiota [38]. Research has shown that FOS and GOS can be metabolized by some *Lactobacillus* strains and that they promote the colonization process [39]. In this process, a series of small metabolites (such as SCFAs) are produced [40], and the proliferation of intestinal cells is promoted [21]. Thus, the oligosaccharide fermentation ability of strains can help maintain a stable gut environment and strengthen the epithelial barrier, which could contribute to the prevention of IBD. The adhesion ability of strains is a physiological characteristic also related to the IBD-alleviating functions of some strains. A high adhesion ability is advantageous in the intestinal colonization process and could prevent the damage caused by IBD and inhibit pathogenic bacteria adhesion [41]. During the process of adhering to intestinal cells, S-layer proteins react with host receptors to reduce the expression of TNF-α and IL-12 [42] and modulate signaling pathways within the host cells [43]. This is necessary for probiotics to regulate host immune responses and alleviate IBD. Generation time, an essential physiological characteristic of strains, is also a direct indication of the growth rate of a strain. Strains with a fast growth rate and a short generation time can compete for more nutrients in the gut microenvironment, which is beneficial in the relief of intestinal mucosal injury in IBD and resistance to pathogenic bacterial infections. Aside from stress resistance and growth ability, the ability of probiotics to produce beneficial substances, such as EPSs, acetic acid and CLA, is also related to IBD alleviation. EPSs are heterogeneous structures secreted by bacteria that help antagonize pathogenic bacteria [25], promoting the synthesis of SCFAs [44]. In the process of repairing IBD-induced inflammatory damage, EPSs can activate macrophages, promote the proliferation of T cells and inhibit TNF-α expression [45]. In the case of IBD-induced apoptosis, EPSs can regulate the expression of p53 and activate Caspase-3, thus killing tumor cells [46]. SCFAs are small molecules that play an essential role in human health. About 90% of SCFAs in the body are produced by colonic bacterial fermentation. Acetic acid is an SCFA. In vivo, acetic acid can be absorbed by intestinal epithelial cells to provide energy, strengthen gastrointestinal motility and protect the intestinal mucosal barrier [24], thus preventing pathological damage of the colon in IBD. In addition, adequate acetic acid supplementation can inhibit the proliferation of colorectal cancer cells and inhibit tumorigenesis [47]. CLA is a long-chain fatty acid found in humans and animals with many known health functions, such as anti-oxidation [48] and prevention of diabetes [49] and cardiovascular disease [50]. In addition, CLA can also regulate the body’s immune response. Multiple reports indicate that CLA can reduce expression of TNF-α in the body, induce PPARγ receptor expression and inhibit the expression of NF-κB, thereby alleviating IBD symptoms [51, 52]. Therefore, all physiological characteristics of the strains, including stress resistance, growth ability and metabolic ability, are potentially related to their beneficial effects on IBD.

Based on these in vitro indicators, *L. casei* with different physiological characteristics was screened, and its efficacy in alleviating IBD was also evaluated. The results showed that the therapeutic effect of *L. casei* strains on IBD was strain-specific. In animal models, three indicators indicate the severity of IBD directly: body weight, DAI score and colon length. Moreover, most studies suggest that IBD may be related to an overreaction of the immune system to gut microbiota [53]. Therefore, the gut microbiome composition in each group was measured to explore whether each strain could alter the gut microbiome favorably. IL-10 is an anti-inflammatory cytokine that plays a vital role in controlling and preventing IBD. IL-10 is thought to block the metabolism of macrophages and promote impaired mitochondrial autophagy to reduce inflammation [54]. IL-22 is an anti-inflammatory cytokine of the IL-10 family that can induce the phosphorylation of STAT3 in intestinal epithelial cells, accelerate cell proliferation, and promote the recovery of the damaged intestinal mucosa [55]. NF-κB is a family of proteins with multi-directional regulatory functions and can regulate the expression of pro-inflammatory cytokines, immune receptors, and apoptosis-related proteins. p65 is a representative protein of the NF-κB pathway, with the phosphorylation level of p65 being directly related to activation of the signaling pathway [56]. However, the translocation of NF-κB to the nucleus and the DNA binding process of p65 could be inhibited by IL-10, thereby preventing tissue inflammation and apoptosis [57]. Therefore, the expression levels of IL-10, IL-22, and NF-κB in colon tissue were also determined in this study. The results demonstrate that only *L. casei* M2S01 had a therapeutic effect on IBD symptoms. It not only effectively relieved the weight loss and DAI increase in mice with colitis induced by DSS, but also favorably regulated the gut microbiome through increasing the relative abundance of *Bifidobacterium* and decreasing the relative abundance of *Bacteroides*. Furthermore, *L. casei* M2S01 promoted the expression of anti-inflammatory cytokines. Histological evaluation showed that supplementation with *L. casei* M2S01 alleviated colon tissue injury. Western blot results also showed that phosphorylation of p65 in the colon of the M2S01 group was significantly decreased, indicating that the activation of NF-κB was inhibited. This suggests that the beneficial physiological effects of *L. casei* M2S01 may be related to modulation of the NF-κB signaling pathway. Up to this point in this study, *L. casei* M2S01 had been screened as a probiotic strain with IBD-alleviating effects.

*L. casei* M2S01 showed excellent gastrointestinal transit tolerance, suggesting that this ability may be one of the prerequisites for the IBD-alleviating effects of *L. casei*. But *L. casei* M2S01 was not as good in terms of other physiological characteristics. In contrast, *L. casei* VM2, had good stress resistance, excellent growth and EPS synthesis ability, and was presumed capable of alleviating IBD. However, it did not show any therapeutic effect on IBD symptoms in this animal model. Not all *L. casei* strains with good gastrointestinal tolerance ability could alleviate IBD effectively.

There are three main internal factors that could lead to this mismatch. First, *L. casei* M2S01 might have some physiological characteristics or some metabolites that were not considered in this study but which participated in the process of alleviating IBD symptoms. Host-microbe interactions involve various microbial surface molecules and metabolites. Several studies have shown that bacterial surface proteins and secretory proteins have a preventive effect on inflammation. For example, the S-layer protein on the surface of *Lactobacillus acidophilus* can bind to the C-type lectin SIGNR3, regulating the gut microbiota and immune responses to protect the intestinal mucosal barrier, alleviating IBD [58]. p40 and p75, produced by *Lactobacillus rhamnosus* GG, can effectively regulate the activation of epidermal growth factor receptor (EGFR) and Akt, and also inhibit cytokine-induced epithelial cell apoptosis and the pathological damage of IBD [59]. Moreover, indole [60], bacteriocin [61] and other substances metabolized by some probiotic strains also have an inhibitory effect on pathogenic bacteria and could regulate the expression of pro-inflammatory factors like IL-8, reducing inflammation. Second, the synergistic effect of various physiological characteristics may also contribute to *L. casei* M2S01 having an IBD-alleviating phenotype. Probiotics play a health-promoting role in vivo through complex processes in which several physiological attributes may be involved. Studies have shown that *Lactobacillus*-mediated inhibition of colon tumor cell proliferation is associated with *Lactobacillus* adhesion to the tumor cells and the subsequent production of butyric acid [62]. The inhibitory effect of *L. plantarum* on *Salmonella* in vivo may be related to its high gastrointestinal transit tolerance, adhesion ability and pathogen antagonism [63]. So, the alleviating effect of *L. casei* M2S01 on IBD may be attributable to the combined effects of various physiological characteristics. Finally, the environmental differences in vivo and in vitro may lead to different colonization, growth and metabolism of the strains. Compared with the in vivo environment, the in vitro environment is a relatively pure environmental condition. Therefore, the physiological characteristics of probiotics in vitro may be different from those in vivo. According to previous studies, the gastrointestinal transit tolerance of probiotic strains can be affected by the types of carbon sources in the growth environment [64], while CLA synthesis can be limited by the concentration of carbon sources [65]. However, the carbon sources in vitro and in vivo are often different. This may lead to some differences in the ability of the strains to alleviate IBD.

In summary, gastrointestinal transit tolerance ability may be one of the prerequisites for the IBD-alleviating effects but is not a decisive condition for these same effects.

The purpose of this study was to screen *L. casei* strains with IBD-alleviating effects based on the in vitro physiological characteristics. The results showed that *L. casei* M2S01 significantly restored the gut microbiota composition, activated the immune response, and alleviated the symptoms of IBD in a mouse model. A good gastrointestinal tolerance ability may be one of the prerequisites for the IBD-alleviating effects of *L. casei*. Our findings confirm the potential efficacy of *L. casei* for alleviating IBD and lay the foundation for the rapid screening of *L. casei* strain with IBD-alleviating effects.

## Figures and Tables

**Table 1:** FOS and GOS utilization by *Lactobacillus casei* strains.

Strain	FOS	GOS
5-1L	+	/
MJ1	+	/
NT72-1	+	/
M25	/	/
VM3	/	/
V-CQRC7-161-M2	/	+
M2S01	/	/
V-CQQJ4-174-M3	+	/
VM2	+	+
CCFM30	+	+

+ Indicates FOS/GOS was fermented by this strain. / Indicates FOS/GOS was not fermented by this strain. FOS, fructooligosaccharides; GOS, galactooligosaccharides.
